# A self-adaptive attraction and repulsion-based naked mole-rat algorithm for energy-efficient mobile wireless sensor networks

**DOI:** 10.1038/s41598-024-51218-0

**Published:** 2024-01-10

**Authors:** Supreet Singh, Urvinder Singh, Nitin Mittal, Fikreselam Gared

**Affiliations:** 1 Department of ECE, Thapar Institute of Engineering and Technology, Patiala, India; 2 Department of CSE, Faculty of Engineering and Technology, SGT University, Gurugram, India; 3 Skill Faculty of Engineering and Technology, Shri Viswakarma Skill University, Dudhola, Haryana India; 4 Faculty of Electrical and Computer Engineering, Bahir Dar Institute of Technology, Bahir Dar University, Bahir Dar, Ethiopia

**Keywords:** Evolution, Engineering

## Abstract

Naked mole-rat algorithm (NMRA) is a swarm intelligence-based algorithm that draws inspiration from the mating behaviour of mole rats (workers and breeders). This approach, which is based on the ability of breeders to reproduce with the queen, has been utilized to tackle optimization problems. The algorithm, however, suffers from local optima stagnation problem and a slower rate of convergence in order to provide gobal optimal solution. This study suggests attraction and repulsion strategy based NMRA (ARNMRA) along with self-adaptive properties to avoid trapping of solution in local optima. This strategy is utilized to create new breeder rat solutions and mating factor $(\lambda )$ is made self-adaptive using simulated annealing (*sa*) based mutation operator. ARNMRA is evaluated on CEC 2005 numerical benchmark problems and found to be superior to other algorithms, including well-known ones like selective operation based GWO (SOGWO), opposition based laplacian equilibrium optimizer (OB-L-EO), improved whale optimization algorithm (IWOA), success-history based adaptive DE (SHADE) and original NMRA. Further, according to experimental results, the performance of ARNMRA is likewise superior to the NMRA for the CEC 2019 and CEC 2020 numerical problems. Convergence profiles and statistical tests (rank-sum test and Friedman test) are employed further to validate the experimental results. Moreover, this article extends the application of ARNMRA to address the data gathering aspect in mobile wireless sensor networks (MWSNs) with the goal of prolonging network lifetime and enhancing energy efficiency. In this MWSN-based protocol, a sensor node is elected as a cluster head based on factors like mobility, residual energy, and connection time. The protocol aims to maximize the system lifetime by efficiently collecting data from all sensors and transmitting it to the base station. The study emphasizes the significance of considering dynamic node densities and speed when designing effective data-gathering protocols for MWSNs.

## Introduction

A number of gradient-based optimization strategies have been proposed in the recent years by researchers to address a variety of problems. These strategies are found to be competitive, but they have a number of shortcomings. The two main drawbacks are parameter tuning issues and local optima stagnation. These algorithms either stop moving towards the overall optimal solution or get stuck in a particular local optimal solution. Additionally, because the solutions produced by these algorithms are dependent on assumptions, they are ineffective for dealing with computationally expensive problems^1^. The same context led to the development of nature-inspired algorithms (NIA), which were created to overcome the problems with conventional optimization methods. Nature has served as an important source of motivation for humans to overcome difficulties in the real world for millions of years. Due to their speed and adaptability, NIA has become well-known in almost every area of research^2^. The global solution can be found using these population-based techniques without the need for gradient information. Their widespread appeal stems from this essential factor, which does not call for an initial assessment. Evolutionary algorithms (EA) and swarm intelligence (SI) algorithms make up the majority of the many algorithms that have been proposed in this field.

The goal of EA is to identify the most suitable individual among all potential solutions and pass it on to the upcoming generation. This is because there is a higher chance that the best solutions from the previous generation will lead to more optimal solutions. A well-known evolutionary algorithm is the genetic algorithm (GA)^3^ which was developed based on Darwin’s theory of evolution. To address the shortcomings of GA, the differential evolution (DE)^4^ technique was later developed. The other optimization methods in this group are moth flame optimization (MFO)^5^, evolutionary strategy (ES)^6^ and biogeography-based optimization (BBO)^7^.

The social behaviour of swarms of insects such as ants, bees and birds, served as the inspiration for SI, a form of meta-heuristic algorithm. This method is used by swarms to interact with one another and their environment while searching for food or prey. Both self-organization and task division are essential elements of these strategies. Particle swarm optimization (PSO)^8^ mimics the behaviour of flocks of birds. Other algorithms in this category include the cuckoo search (CS)^9^, salp swarm algorithm (SSA)^10,11^, grey wolf optimization (GWO)^12^, artificial rabbits optimization^13^, bat algorithm (BA)^14^ and naked mole-rat algorithm (NMRA)^15^. Recenty introduced optimization algorithms include Diversity-maintained multi-trial vector differential evolution algorithm for non-decomposition large-scale global optimization (DMDE)^16^, Binary approaches of quantum-based avian navigation optimizer to select effective features from high-dimensional medical data^17^, MFO-SFR: an enhanced moth-flame optimization algorithm using an effective stagnation finding and replacing strategy^18^ and Quantum-based avian navigation optimizer algorithm (QANA)^19^ that prove their worth for solving different optimization problems.

NMRA is another recently proposed SI optimization technique that mimics the natural breeding habits of mole-rats. This algorithm requires tuning of only two parameters such mating factor $(\lambda )$ and breeding probability (*bp*). NMRA is used to address some actual optimization issues, including the location of nodes in wireless sensor networks that are^20^ and the design of ultra wide band antennas with DE hybridization^21^. Despite being a competitive algorithm, NMRA suffers from local optimal stagnation problem, which spurs the researcher to create an improved version of the classical NMRA.

In the present work, utilizing both the global worst and global best solutions^22^ to implement the attraction and repulsion strategy in the breeder phase (exploitation phase) of the proposed algorithm named as ARNMRA. In order to find the best solution, this method enables the search agents (breeder rats) to wander arbitrary under the effect of both attraction and repulsion. At this point, the implementation of the global worst solution can help increase population diversity and issue of premature convergence is handled. The main contribution of this paper can be summed up as follows:

- A self-adaptive attraction and repulsion-based NMRA is proposed with improved exploration and exploitation properties.
- The working efficiency of proposed ARNMRA is tested for CEC 2005 and CEC 2019 numerical problems.
- The statistical results of ARNMRA have been compared with other state-of-the-art algorithms and validated by two statistical tests, namely the rank-sum (p-rank) test and Friedman (f-rank) test.
- A clustering protocol for mobile WSN is developed using ARNMRA for optimal selection of CH.
- To achieve an extended stability period, a clustering solution inspired by the optimization method ARNMRA is taken into consideration.

This article is partitioned into various sections, with an introduction to the article is discussed in “Introduction” section and a mathematical model of classical NMRA is presented in “Mathematical model of NMRA” section. The proposed approach for enhancing the effectiveness of the fundamental NMRA is presented in “Proposed algorithm: attraction and repulsion based naked mole-rat algorithm” section and the statistical findings for the CEC 2005 and CEC 2019 numerical benchmark problems are covered in “Results and discussion” section. In “Real time application: energy-efficient ARNMRA based routing protocol (EARNRP) for mobile wireless sensor networks” section details the clustering algorithm for energy efficient mobile WSN. Finally, “Conclusion and future scope” section includes the article’s conclusion and future scope.

## Mathematical model of NMRA

One of the most well-known swarm intelligent meta-heuristic approaches is NMRA, which was proposed by^15^. This method divides the mole-rat population into worker rats and breeder rats based on the swarm intelligence behaviour of mole-rats found in nature. Breeder rats are useful for performing the exploitation phase of the algorithm, whereas worker rats are primarily incharge of doing the exploration phase. These mole-rats often reside in colonies approximately 70 in size, and the queen of each colony serves as its leader. The following phases are used to define the NMRA mathematical model (exploitation).

### Population initialization

The initial distribution of mole-rats (*MR*) in the search space with dimension (*d*), where (*d*) is the variable count in the problem to be optimised. Each mole rat is initialised using the following equation:

1 $$\begin{aligned} MR_{p,q} = MR_{min,q} + rand \times \left( MR_{max,q}-N_{min,q}\right) \end{aligned}$$

where *p* $\varepsilon$ $[1,2,\ldots MR]$, *q* $\varepsilon$ $[1,2\ldots ,d]$, $MR_{p,q}$ presents *p*th solution of mole rat for *q*th dimension, $MR_{min,q}$ and $MR_{max,q}$ specify the lower and upper bounds of objective function. The random number *rand* has a uniform distribution between 0 and 1.

### Exploration phase (worker phase)

In order to increase their chances of becoming breeding rats and eventually mating with the queen, worker rats often improve their fitness during this phase. As a result, the new worker rat generates a solution based on its own past and local information. If the new mole rat’s fitness for mating is superior, the old solution is disregarded, and the new solution is memorized. If not, the previous solution will be applied. The overall fitness of each rat is recorded once they have all finished the search. To determine the solution to the new worker rat’s use this equation:

2 $$\begin{aligned} MW_p^{t+1}=MW_p^t+\lambda \left( MW_u^t-MW_v^t\right) \end{aligned}$$

where $MW_p^t$ designates the *p* thworker’s solution produced in the *t*th iteration, $MW_p^{t+1}$ designates a new worker mole rat’s solution, parameter $\lambda$ corresponds to the factor dealing with queen’s mating and its value is determined at random between 0 and 1, and $MW_u^t$ and $MW_v^t$ are two worker’s solutions chosen at random from the population.

### Exploitation phase (breeder phase)

Breeder mole-rats in this phase must also maintain themselves updated in order to be chosen for mating and to continue to be breeders. Based on the overall initial best solution $(MR_{best})$ and breeding probability (*bp*), the rats in the breeder’s pool are updated. This *bp* defines at random in the [0,1] range. Some breeder rats might not be able to maintain their fitness and might end up in the worker’s group. The breeding rat solution is generated as follows:

3 $$\begin{aligned} MB_p^{t+1}=(1-\lambda )MB_p^t+\lambda \left( MR_{best}-MB_p^t\right) \end{aligned}$$

where $MB_p^t$ is the *p*th breeder’s solution for the *t*th iteration, the $\lambda$ factor regulates the frequency of breeder rats mating with the queen, and additional breeder rats or the solution $MB_p^{t+1}$ has been produced depending on this frequency. The value of *bp* is initially set at 0.5.

## Proposed algorithm: attraction and repulsion based naked mole-rat algorithm

NMRA has drawn the interest of many researchers due to its success in solving a wide range of practical problems. Since it was developed, the basic, linear approach has been utilized to resolve optimization problems, but it still has drawback of trapping in local optimal solution. Because of this, we present an enhanced version of NMRA in this study that is based on the attraction and repulsion strategy^22^. This suggested algorithm also contains a simulated annealing based mutation operation^23^ to confirm the mating factor $(\lambda )$ applicability to NMRA. Here, it is important to make sure that the attraction and repulsion strategy has been used to modify the Eq. (3) and simulated annealing mutation operator is applied to $\lambda$ parameter of the classical NMRA.

### Adoption of attraction and repulsion strategy

The global optimal solution directs the breeder rats movement in the direction of the ideal solution (mating with queen). However, if the solution is trapped in the local optimal and unable to escape, the entire population is likely to stagnate. This proposed strategy ARNMRA uses the attraction–repulsion method to address this issue. Here, breeder rat solution Eq. (3) has been modified with the help of global best and worst solution using the attraction–repulsion principle moving at random due to the effects of attraction and repulsiveness to discover the best optimal solution. The population’s diversity will be dramatically diminished as the algorithm’s iterative process progresses, which will lead to the premature occurrence. The introduction of the global worst solution may contribute to a rise in population diversity. It solves the issue of early convergence and broadens the population’s scope in the local search process. The following equation is used to obtain attraction–repulsion strategy based breeder rat solution:

4 $$\begin{aligned} MB_p^{t+1}=(1-\lambda )MB_p^t+\lambda \left[ \beta _1 \left( MR_{best}-MB_p^t \right) -\beta _2 \left( MR_{worst}-MB_p^t \right) \right] \end{aligned}$$

where $MB_p^t$ is the breeder mole rat solution for the current iteration *t*, $MR_{worst}$ is the global worst position, $MR_{best}$ is the global optimum solution. The values of $\beta _1$ and $\beta _2$ are considered as 0.5 and 0.4 respectively. The incorporation of attraction and repulsion during the mating process in the breeding mole rats within the ARNMRA algorithm leads to several advantages. Firstly, this interaction enhances the influence on the breeder mole rats, allowing them to be more strongly guided towards the global best solution, represented by the queen. By being influenced by both attraction and repulsion, the breeders are able to explore the solution space more effectively, increasing the likelihood of finding better solutions. Secondly, the interaction of attraction and repulsion promotes a higher level of diversity within the population. The attraction component encourages convergence towards promising regions of the search space, while the repulsion component discourages the population from clustering around local optima. As a result, the population maintains a greater diversity of solutions, which is beneficial for avoiding premature convergence and increasing the chances of finding the global optimum.

### Parameter adaptation

The suggested optimization technique ARNMRA heavily relies on the original NMRA mating factor $(\lambda )$. To achieve better results, this parameter must be altered from its default definition in the basic NMRA. As a result, this option has been altered so that no changes at the user level are necessary and that the parameter $\lambda$ is implemented using *sa* based mutation, which yields the best randomization outcomes. The *sa* based mutation technique is carried out using the generalised equation:

5 $$\begin{aligned} \beta _k= \beta _{min}+\left( \beta _{max}-\beta _{min}\right) \times d^{(s-1)} \end{aligned}$$

where *d* is fixed at 0.95 and enhances the convergence speed of the algorithm, $\beta _{max}$, $\beta _{min}$ and *s* are produced at random from values between [0,1].

Overall, the ARNMRA optimization algorithm is developed using attraction and repulsion strategy along with adaption of important parameter $\lambda$. The major purpose of these modifications is to increase mole-rats diversity, which will enhance exploitation properties and convergence activities. The pseudo-code of the proposed ARNMRA is given in Algorithm 1.

**Algorithm 1:**
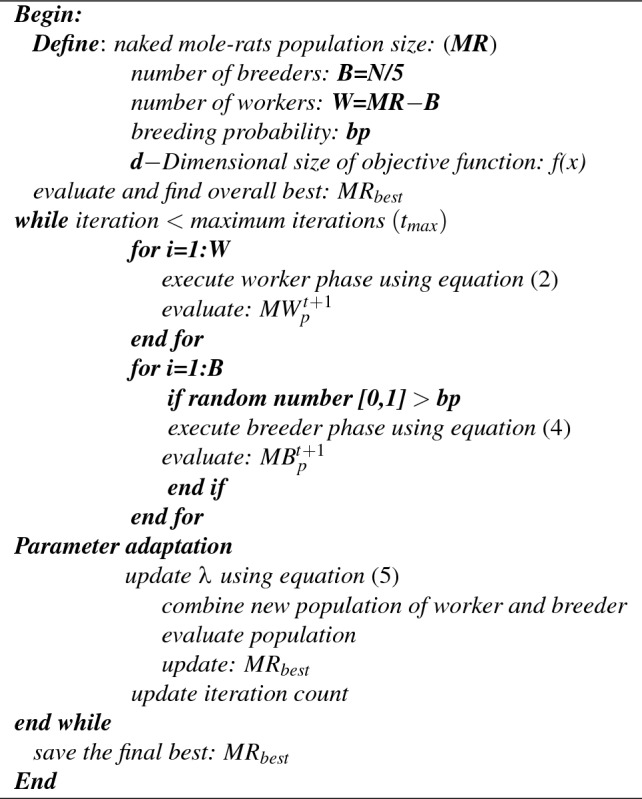
**Pseudo-code of proposed ARNMRA**

## Results and discussion

Three sets of numerical benchmark problems, including the CEC 2005 numerical benchmark problems^24^, CEC 2019^25^, and CEC 2020^26^, are used to evaluate the effectiveness of the proposed ARNMRA. For these test problems, statistical results are produced and are then contrasted with other competitive meta-heuristic methods. The following subsections discuss the statistical outcomes of the proposed optimization technique ARNMRA for these numerical problems.

### Statistical results for CEC 2005 numerical benchmark problems

The statistical findings for the proposed ARNMRA and other competing algorithms are analysed in this subsection. The 12 numerical problems from the CEC 2005 test suite are chosen, and descriptions of these problems are provided in^11^ to evaluate the efficiency of ARNMRA. The numerical problems utilized here can be broadly categorised into two groups: uni-modal problems ($p_1$ to $p_7$) and multi-modal problems ($p_8$ to $p_{12}$). Here, all of the statistical findings are attained with a 30 dimension size, 50 mole-rats population and 500 iterations.

The statistical findings of ARNMRA and various competitive algorithms including traditional NMRA, SHADE, OB-L-EO, SOGWO and IWOA are presented in Table 1. For 51 runs of the algorithm, the statistical findings are presented as mean and standard deviation (std) values. According to these findings, ARNMRA outperforms all other algorithms for problems $p_1$, $p_2$ $p_3$, $p_4$, $p_5$ and $p_7$. SHADE performs the best for problem $p_6$ and $p_{12}$. For numerical problems $p_8$ and $p_{10}$, the four approaches (OB-L-EO, SOGWO, NMRA and ARNMRA) achieve global minimal values While NMRA and ARNMRA provide identical results for $p_9$. In case of problem $p_{10}$, working efficiency of OB-L-EO is found to be superior in comparison with other optimization techniques.

To assess the operational efficiency of ARNMRA, the study employs two statistical tests: the rank-sum (p-rank) test^27^ and the Friedman (f-rank) test^28^. In the p-rank test, the variables *win*(*w*)/*loss*(*l*)/*tie*(*t*) are used to evaluate the performance of ARNMRA compared to other optimization techniques. A “$+$ ”symbol indicates that the compared technique outperforms ARNMRA *win*(*w*), a “−” symbol denotes that it performs worse *loss*(*l*), and “$=$” represents an equal performance *tie*(*t*). The results in Table 1 demonstrate that ARNMRA outperforms the majority of the tested problems. Additionally, the f-rank test assigns a rank to each optimization method being evaluated. The fourth row of Table 1 displays the rank of each technique for each numerical test problem. After assigning f-ranks to all algorithms, the average f-rank value and overall f-rank are calculated (last two rows of Table 1). These results indicate that ARNMRA is a statistically significant optimization technique, consistently ranking at the top among all other algorithms. Here, both the p-rank and f-rank tests confirm the superior performance of ARNMRA. The p-rank test demonstrates that ARNMRA outperforms other techniques in most test problems, while the f-rank test reinforces its statistical significance and consistent top ranking among the evaluated algorithms.

**Table 1:** Simulation results of ARNMRA in comparison with other algorithms for CEC 2005 benchmark test suite.

Problem		SHADE^29^	OB-L-EO^30^	SOGWO^31^	IWOA^32^	NMRA	ARNMRA
$p_1$	Mean	1.421E−09	6.758E−212	6.050E−77	8.131E−146	1.539E−87	**3.246E−292**
	Std	3.090E−09	**0**	1.487E−76	4.358E−145	1.097E−86	**0**
	p-rank	−	−	−	−	−	
	f-rank	6	2	5	3	4	1
$p_2$	Mean	8.700E−03	1.931E−108	1.179E−44	2.379E−102	2.134E−45	**2.155E−150**
	Std	2.132E−02	8.000E−108	1.341E−44	6.577E−102	8.585E−45	**1.164E−149**
	p-rank	−	−	−	−	−	
	f-rank	6	2	5	3	4	1
$p_3$	Mean	1.542E+01	6.931E−187	6.821E−87	1.540E+04	4.503E−104	**6.686E−250**
	Std	9.940E+00	**0**	3.420E−86	7.421E+03	3.029E−103	**0**
	p-rank	−	−	−	−	−	
	f-rank	6	2	4	5	3	1
$p_4$	Mean	9.786E−01	4.732E−103	1.131E−45	1.311E+01	5.526E−45	**8.827E−145**
	Std	7.989E−01	1.600E−102	3.976E−45	1.612E+01	2.212E−44	**6.294E−144**
	p-rank	−	−	−	−	−	
	f-rank	5	2	3	6	4	1
$p_5$	mean	2.441E+01	2.579E+01	2.887E+01	2.651E+01	2.897E+01	**2.176E+01**
	std	1.120E+01	1.556E−01	1.879E−02	6.600E−01	1.840E−02	4.070E−02
	p-rank	−	−	−	−	−	
	f-rank	2	3	5	4	6	1
$p_6$	Mean	**5.311E−10**	9.091E−05	6.776E+00	3.630E−02	6.610E+00	1.510E+00
	Std	**6.352E−10**	5.977E−05	5.788E−01	6.947E−02	5.673E−01	5.178E−01
	p-rank	+	+	−	+	−	
	f-rank	1	2	6	3	5	4
$p_7$	Mean	2.352E−02	4.700E−04	5.932E−04	1.851E−03	6.230E−04	**3.053E−04**
	Std	8.800E−03	3.041E−04	4.957E−04	2.358E−03	5.151E−04	**3.296E−04**
	p-rank	−	−	−	−	−	
	f-rank	6	2	3	5	4	1
$p_8$	Mean	8.531E+00	**0**	**0**	**0**	**0**	**0**
	Std	2.190E+00	**0**	**0**	**0**	**0**	**0**
	p-rank	−	$=$	$=$	$=$	$=$	
	f-rank	6	1	1	1	1	1
$p_9$	Mean	3.952E−01	**8.881E−16**	**8.881E−16**	3.731E−15	**8.881E−16**	**8.881E−16**
	Std	5.857E−01	**0**	**0**	2.168E−01	**0**	**0**
	p-rank	−	$=$	$=$	−	$=$	
	f-rank	6	1	1	5	1	1
$p_{10}$	Mean	4.800E−03	**0**	**0**	2.641E−03	**0**	**0**
	Std	7.700E−03	**0**	**0**	1.100E−02	**0**	**0**
	p-rank	−	$=$	$=$	−	$=$	
	f-rank	5	1	1	6	1	1
$p_{11}$	Mean	3.458E−02	**6.287E−06**	5.611E−02	9.300E−03	1.118E+00	1.295E−01
	Std	8.751E−02	**4.351E−06**	1.421E−02	2.561E−02	2.598E−01	9.180E−02
	p-rank	+	+	+	+	−	
	f-rank	3	1	4	2	6	5
$p_{12}$	Mean	**7.321E−04**	3.832E−02	3.531E−01	1.600E−01	2.991E+00	8.406E−01
	Std	**2.800E−03**	9.751E−02	1.276E−01	1.365E−01	4.150E−02	3.230E−01
	p-rank	+	+	+	+	−	
	f-rank	1	2	4	3	6	5
*w*/*l*/*t*		3/9/0	3/6/3	2/7/3	3/8/1	0/9/3	
f-rank value		4.41	2.00	3.50	3.83	3.75	1.91
Overall f-rank		6	2	3	5	4	1

Significant values are in [bold].

**Figure 1:**
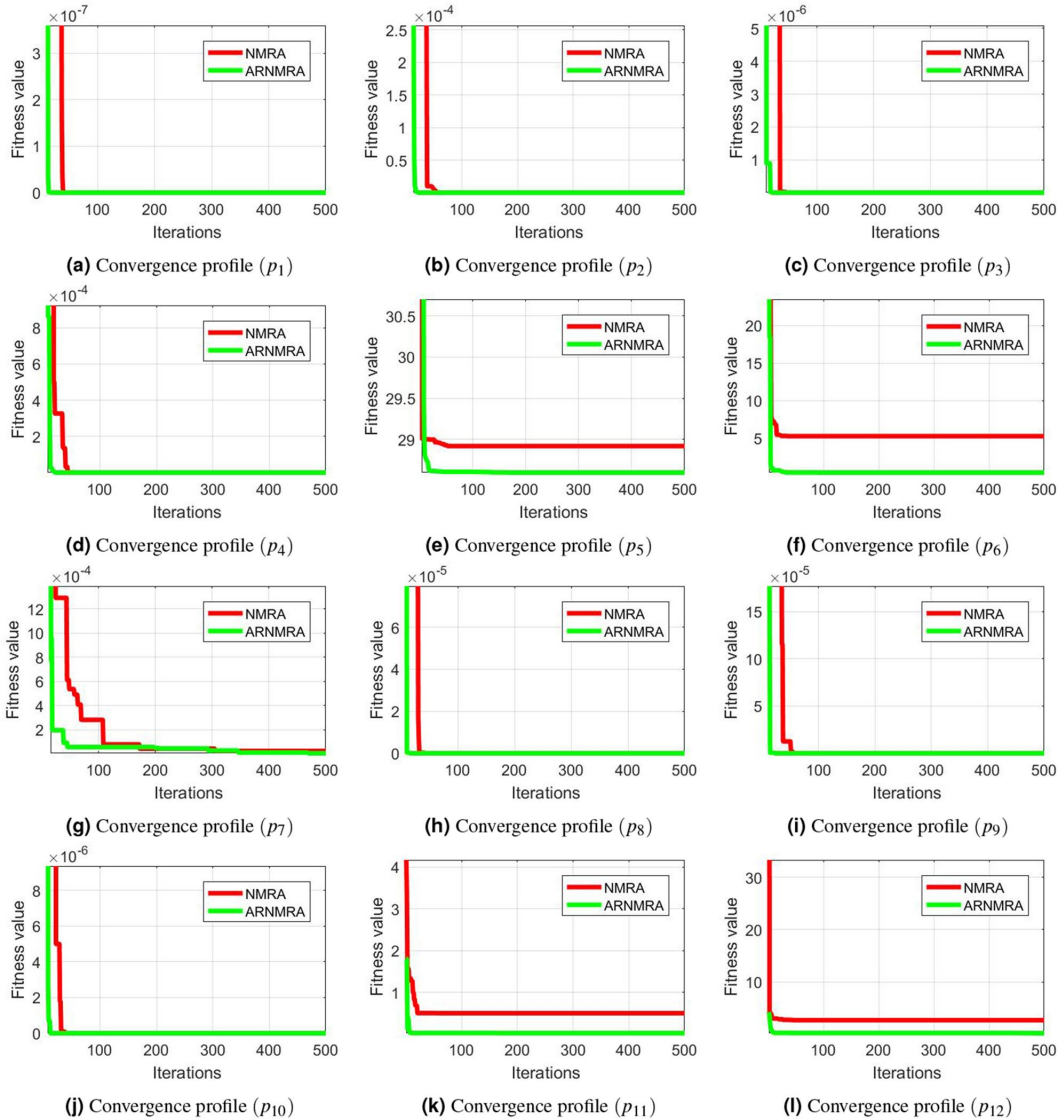
Convergence profiles of NMRA and ARNMRA for CEC 2005 numerical benchmark problems.

For each numerical problem in the CEC 2005 test suite, convergence profiles of the basic NMRA and purposed ARNMRA are also drawn after the simulated results. The convergence graphs shown in Fig. 1 confirm that the proposed ARNMRA converges to the optimal solution more quickly than the conventional NMRA.

### Statistical results for CEC 2019 numerical benchmark test suite

In this subsection, the proposed ARNMRA’s performance is evaluated for the 100-digit challenge (CEC 2019). This test suite contains 10 numerical problems, and an explanation of every test problem can be found in the reference^25^. Here, statistical findings for 51 runs of the algorithms for the original NMRA and the suggested ARNMRA are presented as average, best, median, worst, and standard deviation (std) values. The number of mole-rats should be set to 50, the problem’s dimension should match the description, and a maximum iterations of 500 should be used for generating the results.

Table 2 contains the statistical findings produced for these numerical benchmark functions. For the benchmark problem $b_1$, ARNMRA has a better operating capacity than classical NMRA. The suggested technique ARNMRA is the best for all performance measures for the benchmark problems $b_2$, $b_4$, $b_5$, $b_6$, $b_8$ and $b_{10}$ test functions. For problem $b_3$, the results are compared for standard values and ARNMRA provides the best performance. In case of problems $b_7$ and $b_9$, NMRA’s performance is better as compared to proposed ARNMRA. Overall, it is determined that the suggested optimization technique ARNMRA is shown superior performance for majority of these numerical benchmark problems.

**Table 2:** Simulation results for CEC 2019 numerical benchmark problems.

Problem	Algorithm	Best	Median	Mean	Worst	Std
$b_1$	NMRA	8.320E+04	2.551E+05	3.221E+05	8.397E+05	2.071E+05
	ARNMRA	5.003E+04	1.168E+05	1.350E+05	4.848E+05	7.839E+04
$b_2$	NMRA	1.759E+01	1.858E+01	1.855E+01	1.971E+01	6.112E−01
	ARNMRA	1.739E+01	1.778E+01	1.784E+01	1.866E+01	3.432E−01
$b_3$	NMRA	1.270E+01	1.270E+01	1.270E+01	1.270E+01	2.956E−07
	ARNMRA	1.270E+01	1.270E+01	1.270E+01	1.270E+01	3.245E−08
$b_4$	NMRA	9.163E+01	3.663E+02	4.741E+02	1.636E+03	3.660E+02
	ARNMRA	9.600E+01	3.014E+02	3.528E+02	1.297E+03	2.361E+02
$b_5$	NMRA	1.331E+00	1.978E+00	1.985E+00	2.624E+00	2.792E−01
	ARNMRA	1.407E+00	1.939E+00	1.939E+00	2.589E+00	3.127E−01
$b_6$	NMRA	9.074E+00	1.121E+01	1.115E+01	1.255E+01	8.049E−01
	ARNMRA	9.124E+00	1.039E+01	1.036E+01	1.179E+01	6.533E−01
$b_7$	NMRA	− 1.243E+02	1.981E+02	1.533E+02	4.042E+02	1.227E+02
	ARNMRA	− 2.958E+02	1.019E+01	− 2.860E+00	2.191E+02	1.192E+02
$b_8$	NMRA	4.974E+00	6.003E+00	5.919E+00	6.534E+00	3.304E−01
	ARNMRA	4.565E+00	5.663E+00	5.594E+00	6.151E+00	3.227E−01
$b_9$	NMRA	3.079E+00	4.179E+00	4.354E+00	1.016E+01	1.041E+00
	ARNMRA	3.061E+00	5.180E+00	1.126E+01	1.097E+02	1.932E+01
$b_{10}$	NMRA	1.951E+01	2.049E+01	2.048E+01	2.065E+01	1.628E−01
	ARNMRA	2.017E+01	2.037E+01	2.037E+01	2.057E+01	7.670E−02

**Figure 2:**
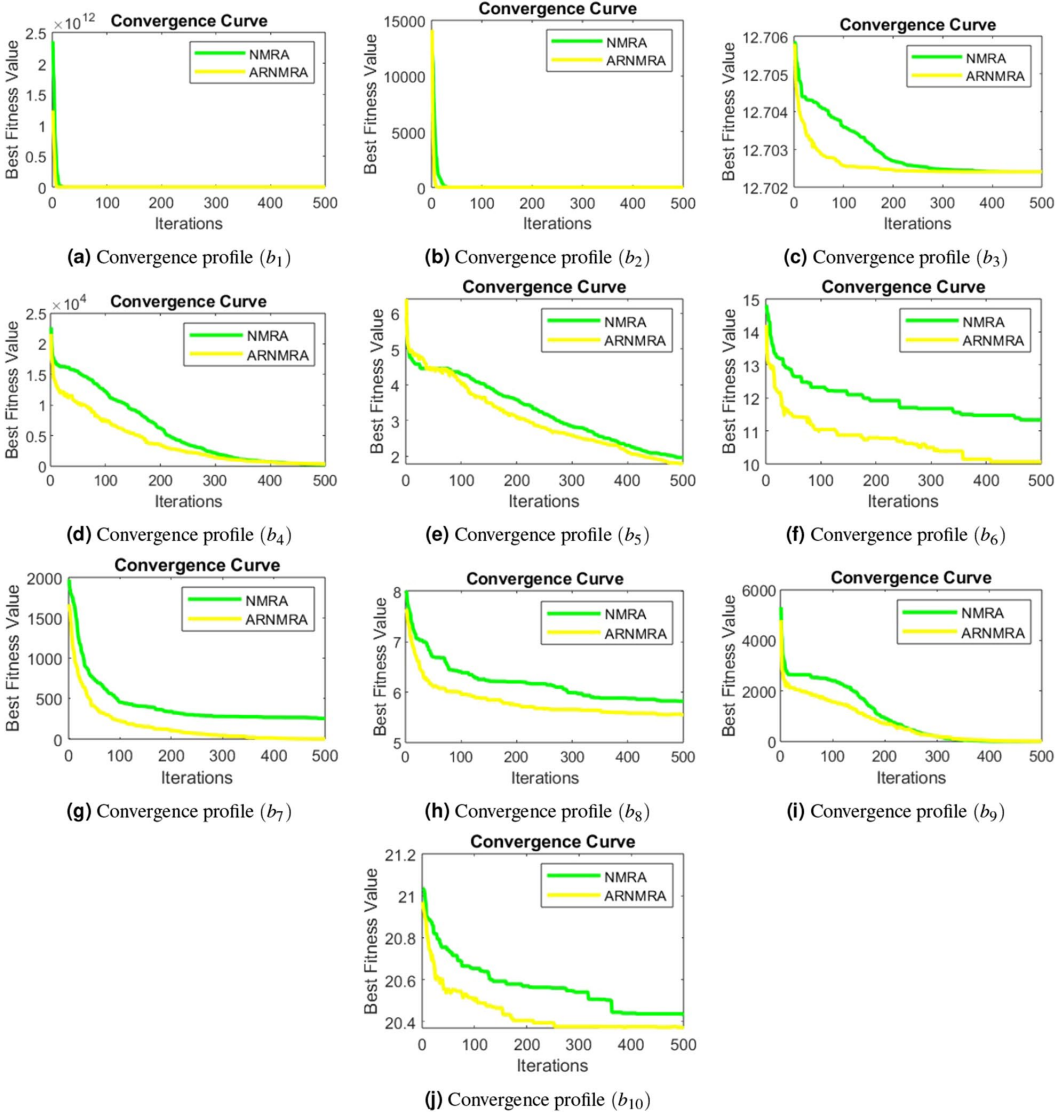
Convergence profiles of NMRA and ARNMRA for CEC 2019 numerical problems.

After obtaining simulated results for each numerical problem in the CEC 2019 test suite, convergence profiles for both the basic NMRA and the proposed ARNMRA are depicted. The convergence graphs, as illustrated in Fig. 2, demonstrate that the ARNMRA converges to the optimal solution at a faster rate compared to the original NMRA.

### Statistical results for CEC 2020 numerical benchmark test suite

The performance of the proposed ARNMRA has been accessed for CEC 2020 test suite in this subsection. This test suite comprises 10 numerical problems, each with detailed explanations available in the referenced work^26^. The statistical results for 51 runs of both the original NMRA and the proposed ARNMRA are presented, including mean, best, worst and standard deviation (*Std*) values. The parameter configuration involves setting the function evaluations $10^4 \times D$, ensuring the problem’s dimension (*D*) is 20 for result generation.

The statistical results presented in Table 3 highlight the performance of the ARNMRA on various numerical test problems. In the case of numerical problems $t_1$, $t_4$ and $t_5$, ARNMRA exhibits superior operational capabilities compared to the classical NMRA. Notably, the proposed technique, ARNMRA, outperforms classical NMRA across all performance metrics for benchmark problems $t_2$, $t_3$, $t_7$, $t_8$, $b_9$, and $t_{10}$. Conversely, for problem $t_6$, NMRA performs better than the proposed ARNMRA. The suggested optimization technique, ARNMRA, demonstrates superior performance for most of these numerical test problems.

**Table 3:** Simulation results for CEC 2020 numerical test problems.

Problem	Algorithm	Best	Mean	Worst	Std
$t_1$	NMRA	6.443E+02	2.444E+03	9.561E+03	1.838E+03
	ARNMRA	2.960E+02	7.197E+02	9.956e+02	2.087E+02
$t_2$	NMRA	5.611E+02	8.675E+02	1.232E+03	1.751E+02
	ARNMRA	4.718E+02	7.848E+02	9.462E+02	1.548E+02
$t_3$	NMRA	6.795E+01	1.350E+02	1.792E+02	2.308E+01
	ARNMRA	8.612E+01	1.095E+02	1.311E+02	1.517E+01
$t_4$	NMRA	6.300E+00	1.115E+01	1.379E+01	2.104E+00
	ARNMRA	8.133E+00	1.060E+01	1.417E+01	1.798E+00
$t_5$	NMRA	6.716E+03	3.966E+04	9.197E+04	2.341E+04
	ARNMRA	9.335E+03	2.980E+04	6.069E+04	1.684E+04
$t_6$	NMRA	2.269E+02	2.268E+02	2.268E+02	9.245E−13
	ARNMRA	4.491E+02	4.491E+02	4.491E+02	4.769E−13
$t_7$	NMRA	3.775E+03	1.064E+04	1.961E+04	4.588E+03
	ARNMRA	2.973E+03	6.344E+03	9.450E+03	2.044E+03
$t_8$	NMRA	1.041E+02	5.513E+02	2.621E+03	9.240E+02
	ARNMRA	1.000E+02	3.097E+02	2.359E+03	6.798E+02
$t_9$	NMRA	1.014E+02	2.008E+02	6.776E+02	1.745E+02
	ARNMRA	1.011E+02	1.395E+02	2.105E+02	5.193E+01
$t_{10}$	NMRA	3.993E+02	4.021E+02	4.060E+02	2.301E+00
	ARNMRA	3.992E+02	4.033E+02	4.066E+02	3.248E+00

## Real time application: energy-efficient ARNMRA based routing protocol (EARNRP) for mobile wireless sensor networks

Wireless sensor networks (WSNs) consist of compact, energy-efficient sensor nodes that communicate without the need for wired connections. These nodes are designed to monitor and gather data from the surrounding physical environment using wireless communication technology^33^. These networks are typically used for applications such as environmental monitoring, industrial automation, healthcare, and smart cities. Mobile WSNs (MWSNs) introduce additional challenges compared to traditional static WSNs due to the mobility of sensor nodes. The movement of sensor nodes introduces dynamic changes in network topology, which can disrupt network connectivity and routing paths. Nodes may join or leave the network, leading to frequent topology reconfigurations. Efficient mechanisms for node tracking, localization, and adaptability to node mobility are required. In MWSNs, sensor nodes are typically powered by batteries, and energy efficiency is crucial for prolonging the network’s lifetime. However, node mobility can cause energy imbalances due to varying distances, resulting in some nodes depleting their energy faster than others. Strategies for energy-efficient routing, power management, and dynamic energy replenishment become essential.

Maintaining connectivity in a mobile environment is challenging. Nodes may move out of each other’s communication range, leading to link failures and frequent disconnections. Reliable and robust communication protocols need to be employed to handle intermittent connections, link quality variations, and node mobility-induced network partitions. Mobile sensor nodes often generate large volumes of data. Efficient data fusion and aggregation techniques are required to reduce redundancy and minimize the amount of data transmitted. However, due to node mobility, data fusion becomes more challenging as nodes move in and out of each other’s sensing ranges. Routing in MWSNs becomes complex due to dynamic topology changes caused by node mobility. Traditional routing protocols designed for static WSNs may not be suitable. Adaptive routing algorithms that can dynamically adjust to changing network conditions, consider node mobility, and provide efficient path planning are necessary. Scalability is a concern in MWSNs, especially when large numbers of mobile nodes are involved. Efficient protocols and mechanisms are needed to handle the increased network size, dynamic topology changes, and frequent node movements without sacrificing network performance and resource utilization.

Addressing these challenges requires the development of specialized algorithms, protocols, and system designs tailored for Mobile WSNs. Researchers continue to explore innovative solutions to overcome these obstacles and unlock the full potential of mobile sensing applications.

Clustering in sensor networks is utilized to reduce energy consumption. However, existing clustering protocols for mobile sensor nodes encounter difficulties in maintaining energy efficiency because they do not adequately consider node movement after clustering. While mobile sensor nodes can offer improved network coverage and connectivity compared to static nodes, effectively managing their operation in line with specific application requirements is a complex task. In various mobile scenarios, existing clustering protocols for mobile sensor nodes often struggle to adequately tackle the challenges related to energy efficiency. One of the key reasons behind this is their failure to account for node movement after clustering. To address the energy efficiency challenges in mobile scenarios, it becomes crucial to develop clustering protocols that can adapt to node mobility. These protocols should take into consideration the movement patterns of nodes and incorporate energy-efficient strategies. By incorporating both node mobility and energy constraints into the design of clustering protocols, it becomes possible to develop more effective solutions that significantly enhance the energy efficiency of mobile sensor networks.

The LEACH-M protocol, an extension of the LEACH protocol^34^, has been specifically designed to accommodate the mobility of sensor nodes. It maintains the same cluster head (CH) formation and selection process as LEACH but introduces membership declaration to ensure the inclusion of sensor nodes during the steady-state phase. LEACH-M improves the rate of successful packet delivery but at the cost of increased control overhead. E-LEACH, an enhanced version of LEACH^35^, incorporates the remoteness mobility metric in its protocol. It aims to enhance the performance of LEACH by considering node mobility. By taking into account node mobility, E-LEACH strives to optimize the routing decisions and improve the overall efficiency of the protocol. CBR^36^ is another protocol proposed for mobile sensor nodes. CBR utilizes adaptive TDMA scheduling and employs round-free cluster heads. This allows the cluster head to receive data not only from its cluster members but also from nodes that enter the cluster. CBR aims to increase the packet delivery rate by enabling efficient data collection within the clusters. While LEACH-M, E-LEACH, and CBR all demonstrate improvements in terms of packet delivery rate, it is important to note that these enhancements come at the expense of increased control overhead and higher energy consumption. These trade-offs must be carefully considered when selecting a clustering protocol for mobile sensor networks.

This section is organized as follows: in “Radio model” section provides an overview of the challenges and requirements in wireless sensor networks with mobile nodes. In “Random waypoint mobility model” section describes the mobility model used in the study. This sub-section explains the characteristics and behaviour of the wireless communication and mobility of sensor nodes. In “System assumptions” and “Protocol description” sections present the details of the proposed protocol, including its design, operation, and key features. This section explains how the protocol addresses the challenges and improves energy efficiency in WSNs with mobile nodes. In “CH election in proposed EARNRP protocol” section deals with CH selection using EARNRP protocol. In “Operation of proposed protocol” section, the proposed protocol’s operation has been discussed. The simulation setup and methodology used to evaluate the performance of the proposed protocol in “Analysis of results” section. It discusses the experimental results, performance metrics, and comparisons with other protocols. The section provides analysis and discussions on the effectiveness of the proposed protocol.

### Radio model

**Figure 3:**
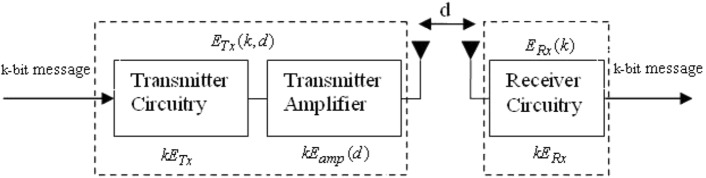
Radio communication model.

The first-order radio communication model^37^ is a widely used model in Wireless Sensor Networks (WSNs) to estimate energy consumption during communication. It considers the energy dissipated by the radio electronics and power amplifier when transmitting a packet from a sender to a receiver. The energy consumption is influenced by factors such as the packet size, distance between the sender and receiver, path loss, and fading effects. Figure 3 illustrates the components involved in the energy dissipation process. To characterize signal propagation in wireless communication, two commonly used models are the free space model and the multipath fading model. These models define how the signal strength and quality vary with the transmitter and receiver separation (*d*). The free space model assumes that the signal propagates through a clear and unobstructed space, whereas the multipath fading model accounts for the effects of reflections, diffractions, and scattering caused by objects and the environment. By utilizing these models, the first-order radio communication model enables the estimation of energy consumption based on the distance between communicating nodes and other relevant factors. It serves as a valuable tool for assessing and optimizing energy efficiency in WSNs.

In the free space model, the assumption is made that wireless signals propagate through unobstructed space without encountering obstacles or interference. This model is typically applicable when the distance between the transmitter and receiver is relatively small, below a certain threshold distance ($d_0$). According to the free space model, the received signal power diminishes in proportion to the square of the distance, as described by the inverse square law.

On the other hand, the multipath fading model considers the effects of signal reflections, diffractions, and scattering caused by obstacles in the propagation environment. It is used for longer distances where the signal experiences multiple paths due to reflections and diffractions. The multipath fading model considers the constructive and destructive interference of these multiple signal paths, resulting in fluctuations in the received signal power. By using these models based on the transmitter and receiver separation, wireless communication systems can better understand and adapt to the characteristics of the propagation environment, leading to improved performance and reliability.

The energy consumed for transmission ($E_{Tx}$) using the above said radio model can be calculated using the following equation:

6 $$\begin{aligned} E_{Tx}(d) = E_{elec}(k)+ E_{amp}* k * d \end{aligned}$$

where $E_{elec}$ is the energy dissipation of the radio electronics to run the transmitter and receiver circuitry. It is a device-specific parameter and is set to 50 nJ/bit [1]. $E_{amp}$ is the transmit amplification energy, *k* is the packet size in bits, and *d* is the sender and receiver separation.

It’s important to note that Eq. (7) only accounts for the energy consumption of the receiver circuitry, as the receiver does not require amplification energy for reception.

7 $$\begin{aligned} E_{Rx} (k)=E_{elec}* k \end{aligned}$$

The first-order radio communication model is a simplified representation of the energy consumption in WSNs and does not capture all the complexities of real-world wireless communication. However, it provides a useful approximation for estimating energy consumption in WSNs.

### Random waypoint mobility model

This mobility model is an extensively employed model for simulating node movement in wireless sensor networks (WSNs). It is widely recognized and adopted due to its ability to provide a realistic depiction of the mobility patterns of nodes within the network

**Figure 4:**
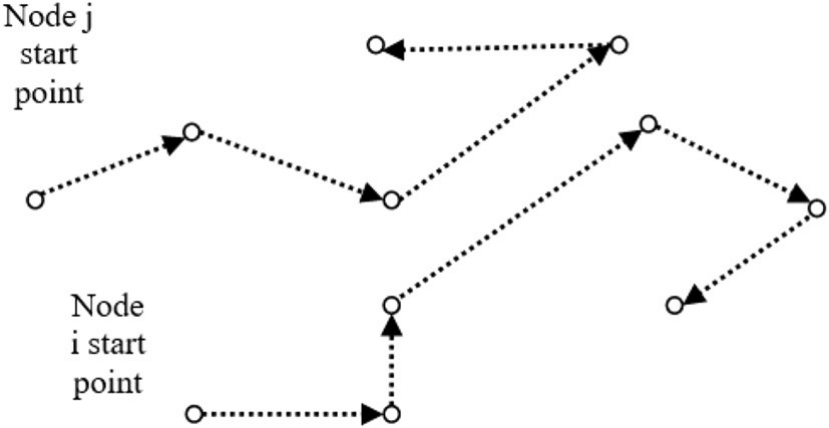
Random waypoint model.

In the random waypoint model^38^, each node in the network follows a random movement pattern. The nodes have a predefined simulation area or region in which they can move. The model assumes that nodes pause for a fixed duration at specific locations and then select a random destination and speed to move towards. Once they reach the destination, they pause again and repeat the process for the duration of the simulation as shown in Fig. 4.

The key parameters in the random waypoint model include:

Pause time ($T_p$): The duration for which a node remains stationary at a particular location before selecting a new destination.

Minimum velocity ($V_{min}$) and maximum velocity ($V_{max}$): The range of speeds at which nodes can move between different locations in the simulation area.

Direction range: The possible range of directions in which nodes can move. Typically represented as an angle or a range of angles (e.g., between 0 and $2{\pi }$).

These parameters govern the movement behavior of nodes in the random waypoint model, allowing researchers to study various aspects of WSNs, such as network connectivity, routing protocols, and energy consumption. By using the random way point mobility model, researchers can evaluate the performance of WSNs under realistic node movement scenarios and develop strategies to optimize network protocols and algorithms to better adapt to dynamic environments.

In the context of our proposed work, we establish several assumptions concerning the random waypoint mobility model. These assumptions form the basis for our research and guide our approach in simulating node movement:

- The simulation area is two-dimensional.
- $P_s$ represents the percentage of static nodes in the network.
- $T_p$ denotes the pause time for each node.
- $V_{min}$ and $V_{max}$ correspond to the minimum and maximum velocities of each individual node, respectively. These parameters determine the range within which the velocities of the nodes can vary during simulation.
- The direction of each node lies within the range of 0 to $2{\pi }$, covering a full circle of possible directions.

### System assumptions

The proposed clustering protocol assumes the following:

Homogeneous and mobile sensors: The sensors in the network are homogeneous, meaning they have the same physical characteristics and capabilities. Additionally, these sensors are mobile, capable of movement within the network.

Location and velocity awareness: Each sensor in the network has awareness of its own location and velocity. This information is crucial for making informed decisions during the clustering process.

Time synchronization: Sensor nodes in the network are assumed to be time synchronized with each other. Time synchronization ensures coordinated operations and facilitates efficient communication among the sensors.

Transmission time calculation: Each sensor is capable of calculating the time it takes for data transmission. This information is utilized during the clustering process and other communication activities within the network.

Single stationary sink: A single sink node is deployed at the center of the network, and it remains stationary. The sink acts as a central point of data collection and aggregation for the sensor nodes.

These assumptions provide a foundation for the proposed clustering protocol, enabling efficient and coordinated operation of the mobile sensor network.

### Protocol description

Existing protocols such as M-LEACH and CBR^39^ consider residual energy for cluster head (CH) election to maximize network lifetime. However, these protocols select CHs based on the minimum distance between nodes, which can lead to packet loss when mobile nodes move out of the cluster. Join request messages are used to mitigate this issue, but they consume additional energy and introduce control overhead.

In order to overcome the challenges mentioned, our proposed protocol, EARNRP, focuses on establishing stable links between cluster heads (CHs) and their member nodes. The protocol aims to improve reliable packet delivery rates by actively searching for stable connections between non-CH nodes and CHs. During the steady state phase, sensor nodes send advertisement messages when they become disconnected from a CH, mitigating packet loss. The selection of new clusters is based on a value associated with each CH, which indicates the suitability of the connection. This value helps in making informed decisions regarding cluster formation. Additionally, after the transmission process, both the CH and its members assess whether a node should remain in the cluster. If a node is deemed unsuitable, the CH removes it from the TDMA (Time Division Multiple Access) schedule and allocates the slot to an alternative node. This approach aims to improve packet delivery rates while minimizing control overhead, thereby enhancing the overall efficiency of the protocol.

### CH election in proposed EARNRP protocol

Unlike the LEACH protocol, which overlooks factors such as remaining energy and node location during cluster head (CH) election, our proposed protocol takes these considerations into account. This is crucial to prevent unbalanced energy consumption and premature depletion of nodes with low residual energy. In our protocol, the CH election process incorporates multiple parameters to make informed decisions. These parameters include the count of previous CHs, the remaining energy of the nodes, the distance between nodes and the sink, the proximity to existing CHs, node mobility, and the duration of their connection time. By considering these factors, we aim to ensure a fair and efficient selection of CHs in the network. Integrating these additional parameters enhances the CH election process, leading to a more balanced distribution of energy consumption and prolonging the overall network lifetime. By factoring in the remaining energy and node location, our proposed protocol addresses the limitations of the LEACH protocol and contributes to improved energy efficiency and longevity of the network.

The combined threshold parameter T(EARNRP(n)) is calculated using the formula:

8 $$\begin{aligned} T_{EARNRP(n)} = {\left\{ \begin{array}{ll} Z(n) &{}\quad if \; E(n) \hspace{2pt} \ge \frac{1}{N} {\sum }_{i=1}^N E(i) \\ 0 &{}\quad if \; E(n) \hspace{2pt} < \frac{1}{N} {\sum }_{i=1}^N E(i) \end{array}\right. } \end{aligned}$$

where

9 $$\begin{aligned} Z(n)=a_1T_1(n)+a_2T_2(n)+a_3T_3(n)+a_4T_4(n)+a_5T_5(n) \end{aligned}$$

is the weighted sum of five sub-threshold parameters, and the weights ($a_1$, $a_2$, $a_3$, $a_4$, and $a_5$) are assigned to balance their contributions. The five sub-thresholds are calculated as follows:

10 $$\begin{aligned} T_1(n)= & {} \frac{V_{max} - v_{n_{current}}}{V_{max}} \end{aligned}$$

11 $$\begin{aligned} T_2(n)= & {} \frac{E_{n_{current}}}{E_{avg}} \end{aligned}$$

12 $$\begin{aligned} T_3(n)= & {} \frac{R_{tran}-d_{ij}}{R_{tran}} \end{aligned}$$

13 $$\begin{aligned} T_4(n)= & {} \frac{\Delta t_{ij}}{t_{frame}} \end{aligned}$$

14 $$\begin{aligned} T_5(n)= & {} \frac{N_{ch}(n)}{N \sum _{i=1}^{N} N_{ch}(i)} \end{aligned}$$

where current speed of the node presented by ($v_{n_{current}}$) and the maximum speed of the node by ($V_{max}$). We also take into account the average energy of the nodes ($E_{avg}$) and the current energy of the specific node ($E_{n_{current}}$). The distance between node *i* and cluster head *j* is denoted as $d_{ij}$, while $R_{tran}$ represents the transmission range of the nodes. To prevent the factor $R_{tran} - d_{ij}$ from exceeding 1, we ensure that it remains within a valid range. Furthermore, we estimate the connection time ($\Delta t_{ij}$) between node *i* and CH *j*. This estimation helps in determining the duration of the connection, which influences the CH selection process. Additionally, we consider the number of rounds that a node has been a CH so far ($N_{ch(n)}$), as well as the alive nodes count in the current round (*N*). These parameters are instrumental in evaluating the history and status of the nodes during CH election. By taking all of these factors into account, our proposed protocol ensures a comprehensive and informed CH election process. It allows for effective selection of CHs based on factors such as node speed, energy levels, distance to the CH, connection time estimation, and historical information of CH participation.

To optimize the parameters such as $a_1$, $a_2$, $a_3$, $a_4$, and $a_5$, which are bounded within the range of [0, 1], we encounter an NP-hard problem. Consequently, evolutionary algorithms are considered the most suitable choice for solving such optimization problems.

In our paper, we employ the ARNMRA algorithm to tackle this optimization problem and obtain optimal results. The fitness function used in the EARNRP protocol is based on multiple thresholds. To evaluate the performance of the proposed algorithm, a multi-objective fitness function is calculated:

Maximize:

15 $$\begin{aligned} Fitness = a_1 T_1 (n)+a_2 T_2 (n)+a_3 T_3 (n)+a_4 T_4 (n)+a_5 T_5 (n) \end{aligned}$$

Subject to:

16 $$\begin{aligned} \sum _{i=1}^{5} a_i=1 \end{aligned}$$

In our proposed fitness function, we incorporate five fixed and equal weighting parameters, denoted as $a_1$, $a_2$, $a_3$, $a_4$, and $a_5$. These parameters are utilized to adjust the relative importance of the five objective sub-threshold parameters within the fitness function.

Equations (10) to (14) are employed to calculate the specific values of these sub-threshold parameters. These equations capture the relationship between the objectives and define the sub-threshold values based on certain criteria or metrics. By incorporating the five weighting parameters and utilizing the sub-threshold equations, our fitness function provides a comprehensive evaluation of the multiple objectives being considered in the optimization problem.

### Operation of proposed protocol

The proposed protocol operates in two phases: the setup and the steady phase.

*Set up phase* During this phase, each node in the network generates a random number within the range of 0 to 1. If the generated random number is less than the adaptive threshold $T_{EARNRP}(n)$, the node declares itself as a CH. The threshold $T_{EARNRP}(n)$ is calculated using Eq. (8), which determines the likelihood of a node becoming a CH. Once a CH is selected, it initiates the broadcasting of an advertisement message to the member nodes in its vicinity. This message contains relevant information such as the CH’s location and velocity. The advertisement is transmitted using the CSMA/CA MAC protocol. Upon receiving the advertisement message, the member nodes assess their options and decide which cluster to join. This decision is based on evaluating the minimum distance between nodes, which is determined by the received signal strength of the advertisement message. To establish a stable link between the CH and its members, and to minimize packet loss and energy consumption, a value is assigned to each CH. This value is calculated using Eq. (9), which indicates the stability of the link.

*TDMA schedule creation* The process of creating a TDMA schedule involves the following steps:

- Advertisement message: When a CH receives advertisement messages from nodes expressing their interest in joining the cluster, it initiates the process of creating a TDMA schedule.
- Determining time slots: The TDMA schedule is prepared based on the number of nodes in the cluster. time slots are allotted for data transmission.
- Sequential transmission: It is assumed that a total of *n* data frames are sent consecutively. Each node, represented by its sequence number $n_i$, initiates transmission at time $(n_{i}+k(1/(p-1)))\tau$, where k ranges from 0 to n. Here, $\tau$ represents the duration of a time slot.
- Order of $\Delta t_{ij}$: The TDMA schedule is organized in increasing order of $\Delta t_{ij}$, which is the time interval between the start of transmission for node *i* and the arrival of data at node *j*. This organization ensures that the constraint $(n_{i}+k(1/(p-1)))\tau$ $\tau \le \Delta t_{ij}$ is satisfied, maximizing the number of successfully transmitted data packets within the cluster.

By following these steps, the TDMA schedule is created in such a way that data transmission among the nodes is coordinated, allowing for efficient utilization of timeslots and maximizing the number of successfully transmitted data packets within each cluster.

*Steady state phase* In the protocol, the following actions and mechanisms are in place to ensure efficient communication and avoid packet loss:

- Data packet loss: If the CH does not receive data packets from a sensor node, it considers those packets as lost. As a result, the CH eliminates the corresponding member node from its TDMA schedule.
- Join request: If member nodes do not receive a data request message from the CH, they send join request messages to CHs in other clusters, expressing their intention to join. This allows member nodes to find alternative clusters to transmit their data.
- ACK message: Once the CH successfully receives a data packet, it sends an acknowledgment (ACK) message to the member nodes, indicating that the data packet was received.
- Cluster join request: Upon receiving a cluster join request message from a member node, the CH sends an advertisement message to that node, similar to the setup phase. This process eliminates the need for membership declarations and reducing overhead.
- Estimated connection time: Both the CH and member nodes maintain information based on estimated connection time. They periodically check whether a node intends to stay in the cluster. If a node is planning to join a new cluster, it sends a join request message to avoid potential packet loss before disconnecting from the current CH. The CH, in turn, removes the membership declaration of the node.

By implementing these actions and mechanisms, the protocol ensures that data packets are not lost due to communication failures. It allows member nodes to switch clusters if necessary and reduces unnecessary overhead by eliminating membership declarations. The use of estimated connection time helps in managing the cluster membership effectively and avoiding packet loss during transitions.

**Figure 5:**
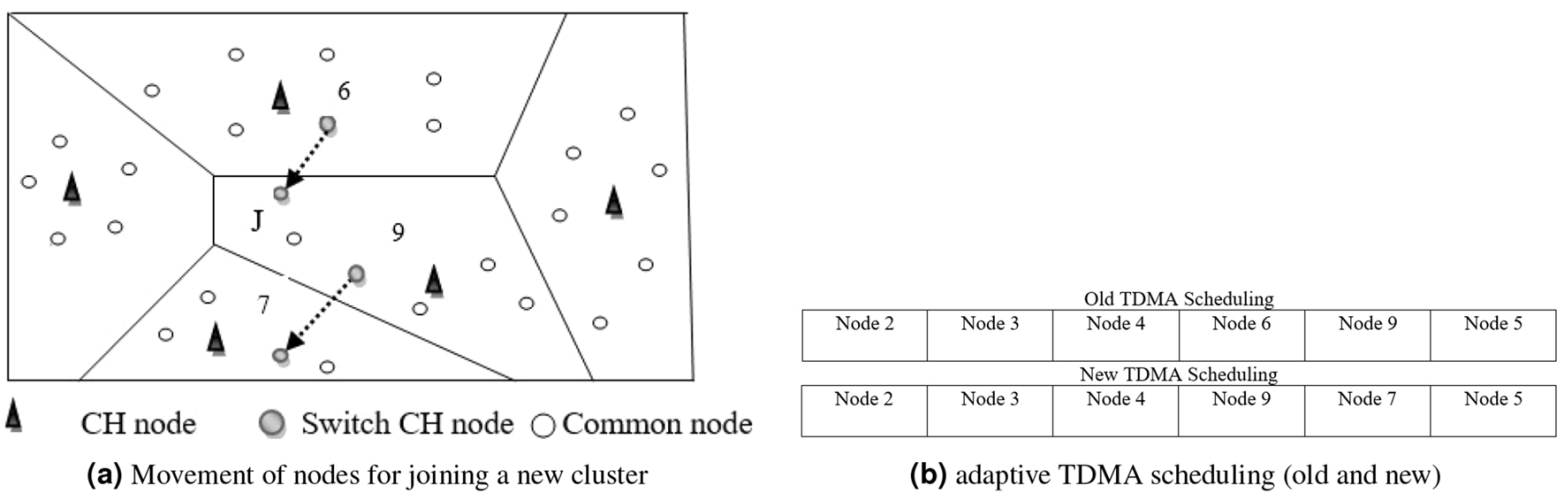
For EARNRP, new adaptive TDMA scheduling creation.

Figure 5a illustrates the process of a node leaving its old cluster and joining a new cluster. In this scenario, Node 9 decides to leave Cluster J, while Node 6 joins the cluster instead. Consequently, the cluster head (CH) of Cluster J modifies the time division multiple access (TDMA) schedule accordingly. Node 6 is included in the schedule, while Node 9 is removed. The adjustment of the TDMA schedule is based on the estimated connection time ($\Delta t_{ij}$), which is organized in ascending order among the member nodes and the CH node. This ordering allows for a systematic arrangement of the nodes within the schedule, ensuring efficient utilization of time slots and effective communication within the cluster.

In Fig. 5b, the updated adaptive TDMA schedule is depicted after the adjustments have been made. Notably, the timeslot previously assigned to Node 9 has been replaced with the timeslot assigned to Node 6. This adaptive TDMA scheduling, facilitated by the adjustments, brings several benefits to the system. Firstly, it improves the rate of successful packet delivery, ensuring that communication between nodes within the cluster is reliable and efficient. By reassigning the timeslot to Node 6, which has joined the cluster, the scheduling enhances the utilization of the channel resources. Furthermore, the adaptive scheduling ensures that the communication resources are efficiently utilized even when nodes join or leave the cluster. This seamless accommodation of node mobility and changes in cluster membership contributes to the overall performance of the system. It allows for effective utilization of available resources while maintaining the desired level of communication quality and efficiency.

### Analysis of results

**Figure 6:**
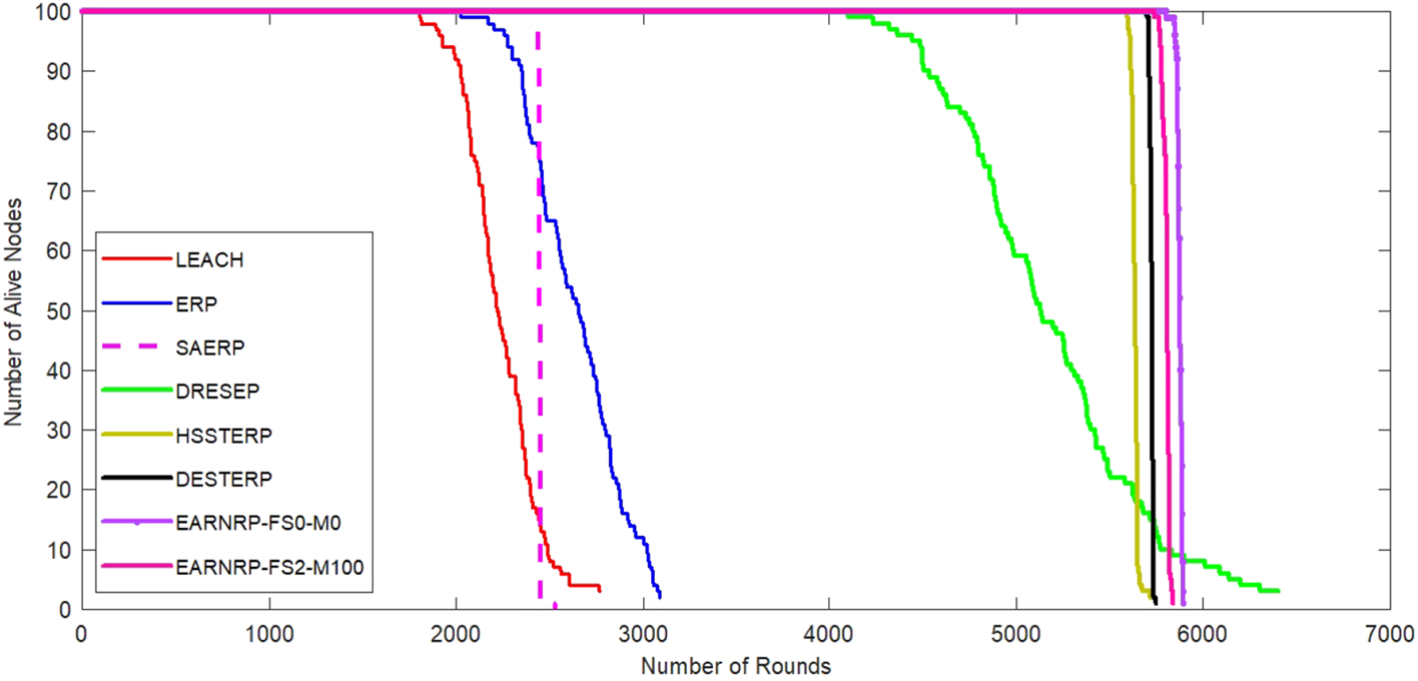
Number of alive nodes as a function of communication rounds for EARNRP in the presence and absence of mobility.

MATLAB is used to analyze the performance of the EARNRP. The simulations are conducted on a network comprising 100 nodes within a 100 m $\times$ 100 m area, with the sink positioned at the center. Figure 6 illustrates the evolution of the count of alive nodes over multiple rounds in all the protocols, excluding the EARNRP protocol, specifically for stationary nodes. This depiction offers valuable insights into the overall behavior and stability of the network nodes over time.

**Figure 7:**
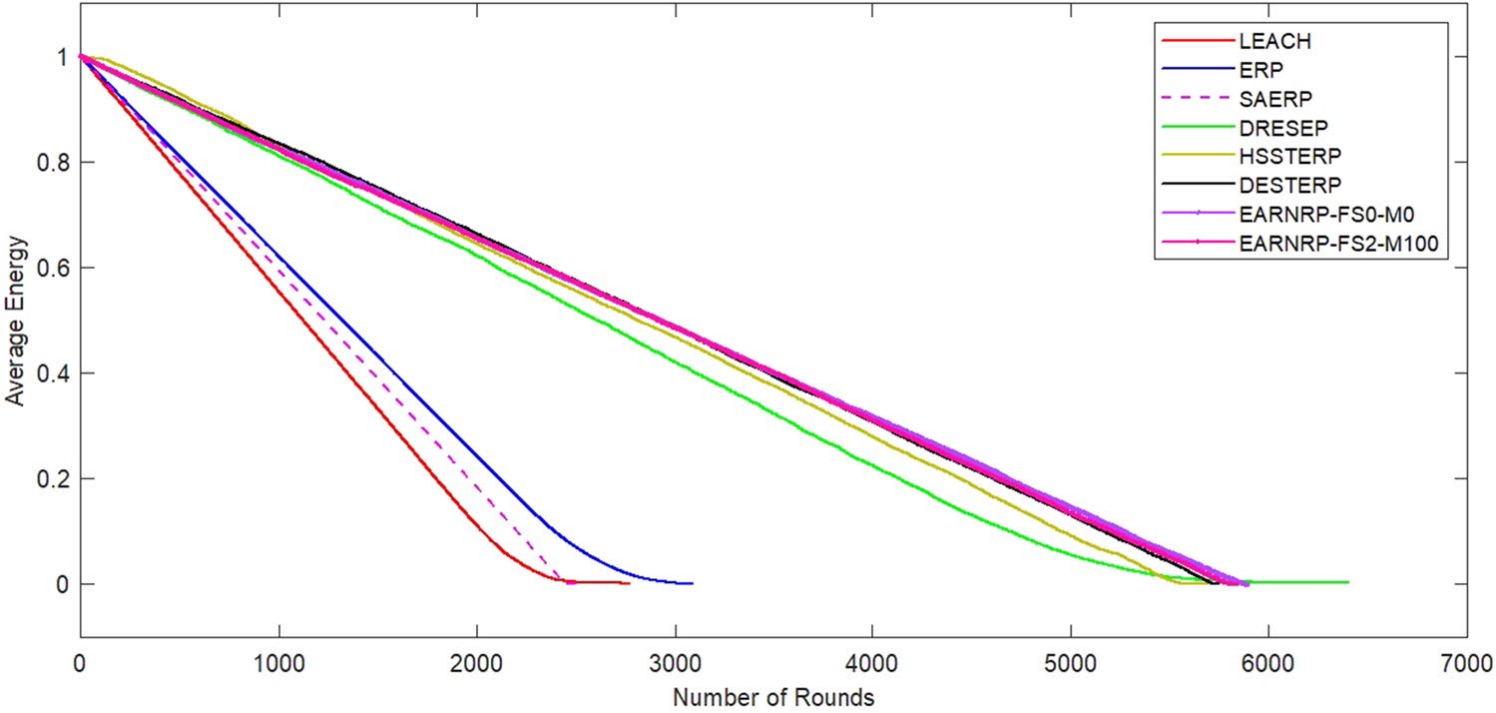
Average energy as a function of communication rounds for EARNRP in the presence and absence of mobility.

In Fig. 6, the alive nodes count is also depicted considering the presence of mobile nodes. The count of alive nodes is measured against the variation in node mobility factor (M) between 20 and 100, and the maximum fixed speed (FS) is set to 2 m/s. Here, the EARNRP with all stationary nodes are designated as EARNRP-FS0-M0 (i.e. EARNRP with fixed speed of 0 m/s and mobility factor 0). Similarly, EARNRP-FS2-M100 represents EARNRP with FS 2 m/s and mobility factor 100). The purpose of this analysis is to estimate the network’s lifetime by observing the alive nodes count in each round. In Fig. 7, we can observe the average remaining energy of the network during the communication rounds. This figure indicates the consumption of overall energy as compared to other competitive methods. Moreover, EARNRP exhibits a consistent and steady energy absorption pattern in each communication round.

To evaluate the performance of the EARNRP protocol against other protocols, several metrics are considered: the first node dies (FND), the half node dies (HND), and the last node dies (LND) as shown in Table 4. These metrics provide insights into the resilience and longevity of the network under different scenarios.

**Figure 8:**
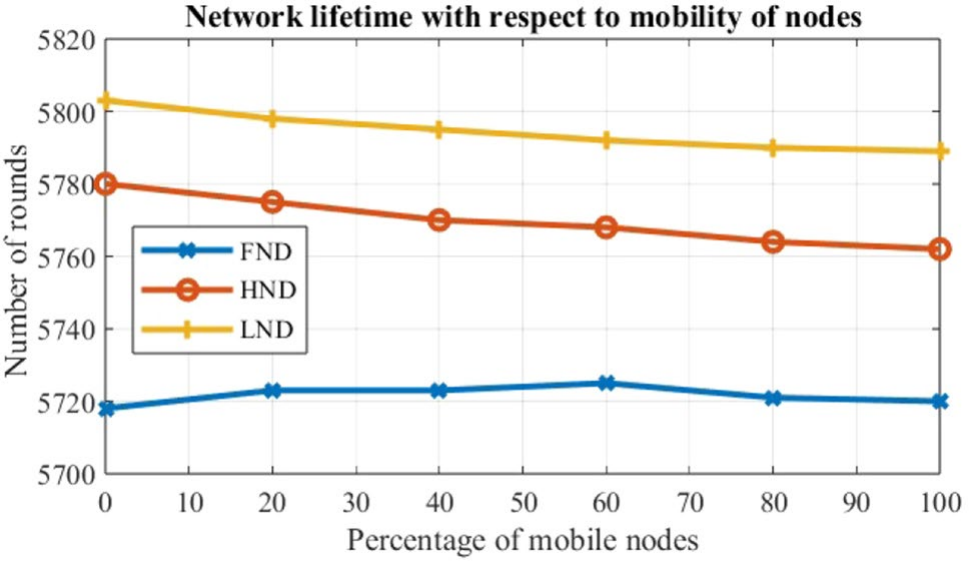
Network lifetime versus percentage of mobile nodes for varying speed.

The research work encompasses three cases for simulations:

Case 1: A fixed speed is assumed while varying node densities. The speed is set to 2 m/s, and simulations are conducted with a dynamic mobility rate ranging from 20 to 100 as shown in Fig. 8. The node count is varied, and its effect on the network’s lifetime is analyzed. Ten simulations are carried out in this scenario, considering a range of 20–100 mobile nodes. The observation reveals that as the mobile nodes count increases, the network lifetime decreases because of the presence of node mobility.

**Figure 9:**
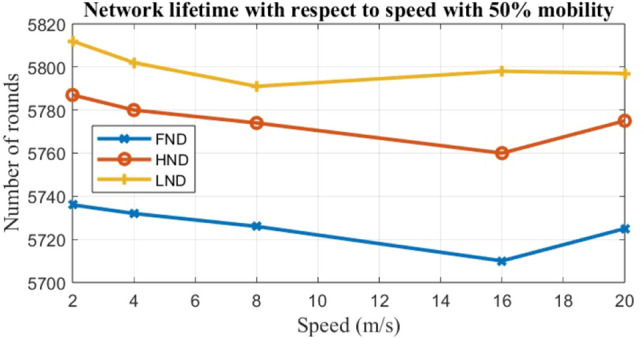
Network lifetime versus speed (m/s) for 50% node mobility.

Case 2: The dynamic speeds are assumed while maintaining fixed mobility, as illustrated in Fig. 9. Ten simulations are conducted with fixed mobility (M = 50) by varying the velocity of nodes from 0.2 to 20 m/s. The analysis focuses on the impact of increasing node velocity on the network’s lifetime.

**Figure 10:**
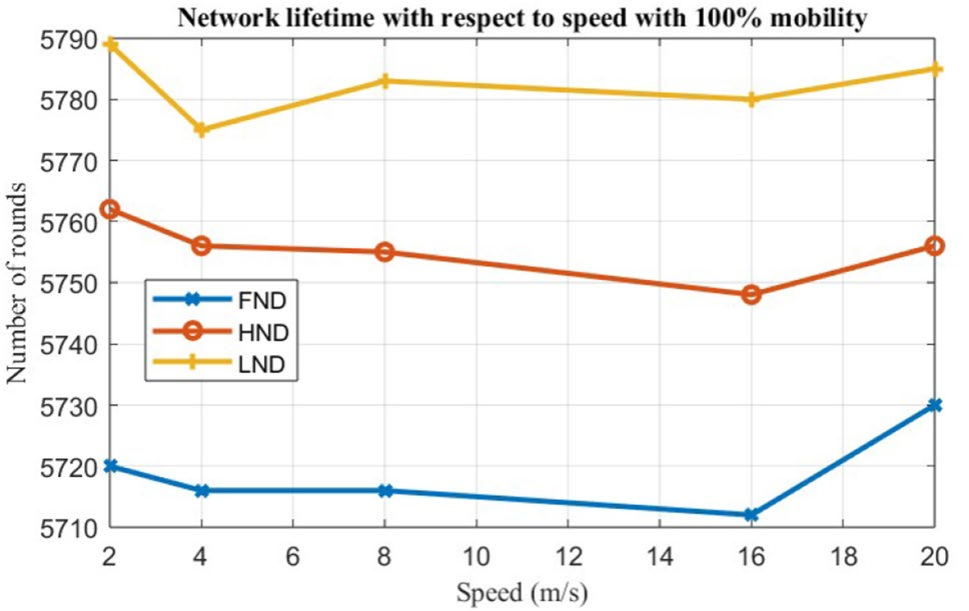
Network lifetime versus speed (m/s) for 100% node mobility.

Case 3: The third case assumes fixed mobility (M = 100) with variation in speeds, as shown in Fig. 10. Ten simulations are conducted with fixed mobility while varying the node count. This scenario considers 100% fixed mobility, and the results are plotted by varying the speed in the range of 0.2–20 m/s.

These three cases provide a comprehensive evaluation of the EARNRP protocol under different scenarios, considering node densities, speeds, and mobility factors. The simulations enable us to assess the network’s performance, lifetime, and stability in various settings.

In the simulations, the performance of the proposed algorithm is evaluated by considering various factors, including the network lifetime. The network lifetime is an important metric that reflects the longevity of the network and its ability to function effectively over time. One of the factors that impact the network lifetime is the percentage of mobile nodes in the network. As the mobile nodes ,count increases, the network becomes more dynamic, which can introduce challenges such as node mobility, connectivity, and energy consumption. The proposed algorithm aims to optimize the network’s performance in the presence of mobile nodes, ensuring efficient resource allocation and minimizing energy consumption to extend the network’s lifetime. Another factor that affects the network lifetime is the speed of the nodes. Higher speeds can result in increased collisions among nodes, leading to network congestion. This congestion can have a negative impact on the network’s performance, reducing its lifetime. The proposed algorithm takes into account the node speeds and dynamically adjusts the network parameters to mitigate congestion and optimize resource allocation, thus improving the network’s lifetime. By conducting simulations and analyzing the network lifetime under different scenarios, the performance of the proposed algorithm has been assessed. The results of these simulations provide insights into the algorithm’s effectiveness in addressing challenges related to mobile nodes, node mobility, and network congestion, ultimately contributing to the improvement of overall network performance and longevity.

**Table 4:** Network lifetime comparison for homogeneous setup with $E_0$=1*J* in terms of dead nodes round history.

Dead Nodes (%)	LEACH	ERP	DRESEP	SAERP	HSSTERP	DESTERP	EARNRP-FS0-M0	EARNRP-FS2-M100
1 (FND)	1805.1	2113.2	4101.9	2437.8	5635.8	5699.2	**5742.9**	5720.6
10	2020.5	2275.8	4503.7	2444.2	5654.6	5711.6	**5779.1**	5726.3
20	2067.8	2364.9	4770.2	2445.4	5667.1	5719.1	**5795.5**	5727.8
30	2140.5	2437.6	4880.5	2447.6	5670.5	5721.8	**5805.3**	5729.2
40	2170.6	2510.3	4984.1	2448.3	5676.3	5722.9	**5810.2**	5734.4
50 (HND)	2213.8	2580.5	5127.0	2449.9	5680.2	5726.3	**5812.9**	5762.3
60	2281.4	2651.2	5293.8	2451.1	5681.9	5727.1	**5815.4**	5780.1
70	2345.8	2745.3	5396.3	2451.8	5688.2	5728.9	**5816.3**	5784.2
80	2393.5	2836.9	5620.9	2452.2	5690.4	5733.1	**5820.2**	5787.8
90	2484.8	2983.8	5771.5	2453.6	5692.8	5735.9	**5823.3**	5788.3
100 (LND)	2763.8	3305.7	**6400.8**	2454.7	5715.9	5738.6	5834.7	5789.6

Significant values are in [bold].

## Conclusion and future scope

This study introduces the attraction and repulsion-based naked mole-rat algorithm (ARNMRA) as an enhancement to the original NMRA. The primary issue with NMRA is its susceptibility to premature convergence and getting trapped in local optima. To overcome this problem, the ARNMRA incorporates an attraction and repulsion strategy along with self-adaptation of the mating factor. The algorithm’s effectiveness is evaluated on benchmark problems from the CEC 2005 and CEC 2019 test suites, and its performance is compared to other algorithms including SHADE, OB-L-EO, SOGWO, IWOA, and NMRA. Furthermore, a mobility-based energy-aware routing protocol (EARNRP) for WSNs with mobile nodes is proposed in this paper. The protocol enables the selection of cluster heads (CHs) based on parameters such as connection time and mobility. Non-CH nodes aim to establish stable connections with CHs during the clustering process, considering the estimated connection time. The TDMA schedule is designed to assign timeslots for data transmission, with the order of timeslots based on the estimated connection time. This scheduling approach ensures efficient utilization of network resources and minimizes collisions among nodes.Simulations are conducted to evaluate the performance of the proposed algorithm and protocol. One key metric analyzed is the network lifetime, which is influenced by factors like the percentage of mobile nodes and varying speeds. Increased mobility and speed can lead to more frequent collisions among nodes, resulting in network congestion. The impact of this congestion on network lifetime and overall performance is investigated. By studying the network lifetime under different mobility and speed conditions, the research provides insights into the effectiveness of the proposed protocol in dynamic network environments. The findings emphasize the importance of considering mobility and speed factors in the design of routing protocols for WSNs with mobile nodes, as they directly impact network performance and longevity.

As a future prospect, the performance of the proposed strategies can be further examined in engineering design optimization problems such as antenna design, robot control, filter optimization, and load dispatch issues. Additionally, the application of the ARNMRA algorithm can be extended to address multi-objective optimization problems, such as feature selection, web-based clustering, and other complex optimization challenges.

## Data Availability

The datasets used and/or analysed during the current study available from the corresponding author on reasonable request.
